# Locomotion-Related Population Cortical Ca^2+^ Transients in Freely Behaving Mice

**DOI:** 10.3389/fncir.2017.00024

**Published:** 2017-04-07

**Authors:** Quanchao Zhang, Jiwei Yao, Yu Guang, Shanshan Liang, Jiangheng Guan, Han Qin, Xiang Liao, Wenjun Jin, Jianxiong Zhang, Junxia Pan, Hongbo Jia, Junan Yan, Zhengzhi Feng, Weibing Li, Xiaowei Chen

**Affiliations:** ^1^Brain Research Center, Third Military Medical UniversityChongqing, China; ^2^Institute of Urinary Surgery, Southwest Hospital, Third Military Medical UniversityChongqing, China; ^3^Department of Psychology, Third Military Medical UniversityChongqing, China; ^4^Brain Research Instrument Innovation Center, Suzhou Institute of Biomedical Engineering and Technology, Chinese Academy of SciencesSuzhou, China; ^5^Clinical Center for Urological Disease, The Third Affiliated Hospital, Chongqing Medical UniversityChongqing, China; ^6^CAS Center for Excellence in Brain Science and Intelligence Technology, Shanghai Institutes for Biological Sciences, Chinese Academy of SciencesShanghai, China

**Keywords:** population Ca^2+^ transients, optical fiber, motor cortex, visual cortex, freely moving mouse

## Abstract

Locomotion involves complex neural activity throughout different cortical and subcortical networks. The primary motor cortex (M1) receives a variety of projections from different brain regions and is responsible for executing movements. The primary visual cortex (V1) receives external visual stimuli and plays an important role in guiding locomotion. Understanding how exactly the M1 and the V1 are involved in locomotion requires recording the neural activities in these areas in freely moving animals. Here, we used an optical fiber-based method for the real-time monitoring of neuronal population activities in freely moving mice. We combined the bulk loading of a synthetic Ca^2+^ indicator and the optical fiber-based Ca^2+^ recordings of neuronal activities. An optical fiber 200 μm in diameter can detect the coherent activity of a subpopulation of neurons. In layer 5 of the M1 and V1, we showed that population Ca^2+^ transients reliably occurred preceding the impending locomotion. Interestingly, the M1 Ca^2+^ transients started ~100 ms earlier than that in V1. Furthermore, the population Ca^2+^ transients were robustly correlated with head movements. Thus, our work provides a simple but efficient approach for monitoring the cortical Ca^2+^ activity of a local cluster of neurons during locomotion in freely moving animals.

## Introduction

Population neural activity in cell groups is thought to contribute to a variety of brain functions, such as sensory information processing (Bullock, 1997; Engel et al., 2001; Griffin et al., 2015), nervous system development (Grosse et al., 2000; Komuro and Kumada, 2005), learning and memory (Engel et al., 2001; Pesaran et al., 2002; Steriade and Timofeev, 2003; Landsness et al., 2009; Rolls et al., 2011), and motor behavior (Churchland et al., 2006, 2010; Shenoy et al., 2013; Erisken et al., 2014). There is now a general consensus that recording Ca^2+^ activity is an effective approach for uncovering the properties and functions of such aggregate neural activity *in vivo* in both neurons, where Ca^2+^ signals allow the inference of spiking activity, and astrocytes, where Ca^2+^ signals mainly indicate activation (Mao et al., 2001; Stosiek et al., 2003). The major advancement in measuring Ca^2+^ signals was the invention and the application of two-photon microscopy in the nervous system (Denk et al., 1990; Yuste and Denk, 1995). Over many years, particularly with the help of the continuous development of Ca^2+^ indicators, two-photon Ca^2+^ imaging has become widely used for detecting neural activities on multiple scales ranging from networks to single synapses in both anesthetized and behaving animals (Stosiek et al., 2003; Chen et al., 2011, 2013; Nadella et al., 2016; Szalay et al., 2016). Another commonly used approach for *in vivo* brain Ca^2+^ imaging is based on the use of charged coupled detector/complementary metal-oxide-semiconductor-based cameras, which are particularly useful for recording large-field Ca^2+^ dynamics in the superficial cortical layers (Berger et al., 2007).

Complementary to two-photon imaging and camera-based large-field imaging, the rapidly developing techniques for recording population Ca^2+^ signals in the deep brain tissues of freely behaving animals include microendoscopic approaches. These require the implementation of optical fibers, fiber-like GRIN lenses or miniaturized head-mounted imaging devices (Grienberger et al., 2012). Along with the current application of cell type-specific labeling of genetically encoded calcium indicators (Hires et al., 2008; Mank and Griesbeck, 2008), these approaches are strongly facilitating our understanding of the contributions of specific neuronal circuits to animal behaviors (Jung et al., 2004; Lütcke et al., 2010; Keller et al., 2012; Ayaz et al., 2013; Ziv et al., 2013; Adelsberger et al., 2014; Jennings et al., 2015; Flash and Bizzi, 2016; Pakan et al., 2016). A particularly simple but efficient method for deep tissue measurements in freely moving animals is the optical fiber-based Ca^2+^ recording approach, also termed fiber photometry (Gunaydin et al., 2014; Guo et al., 2015). This approach has been frequently applied to population recordings of cell bodies, axon terminals and dendrites in neurons (Murayama et al., 2007; Chen et al., 2013; Gunaydin et al., 2014) as well as astrocyte population activity (Schulz et al., 2012; Paukert et al., 2014).

Locomotion is a basic behavior of animals that involves neural activity in many cortical and subcortical networks (Rathelot and Strick, 2009; Levine et al., 2014; Flash and Bizzi, 2016). Among these, the motor and visual cortices have received much attention. The motor cortex has long been shown to play a key role in the planning and execution of voluntary movements (Wise, 1985; Marigold and Drew, 2011; Shenoy et al., 2013). The visual cortex is extensively connected to the motor areas and plays an important role in guiding locomotion (Marigold, 2008). In addition, the firing activity of visual cortex neurons can be altered by locomotion (Niell and Stryker, 2010; Keller et al., 2012; Ayaz et al., 2013). Here, we used the optical fiber-based Ca^2+^ recording approach to monitor population Ca^2+^ signals from layer 5 of primary motor and visual cortices (M1 and V1) during locomotion in freely behaving mice. We reliably observed Ca^2+^ signals preceding locomotion in both regions. Interestingly, M1 signals occurred earlier than the V1 signals. Finally, we found that these population Ca^2+^ signals were highly correlated with head movements.

## Materials and Methods

### Animals

Adult male C57/BL6J mice aged 3–4 months were used for the experiments. The mice were housed in groups, except those mice with implanted optical fibers. The animals had free access to food and water and lived under a 12-h light/day cycle (lights on at 7:00 am). All experimental procedures were performed according to institutional animal welfare guidelines and were approved by the Third Military Medical University Animal Care and Use Committee.

### Optical Fiber Setup

**Figure 1:**
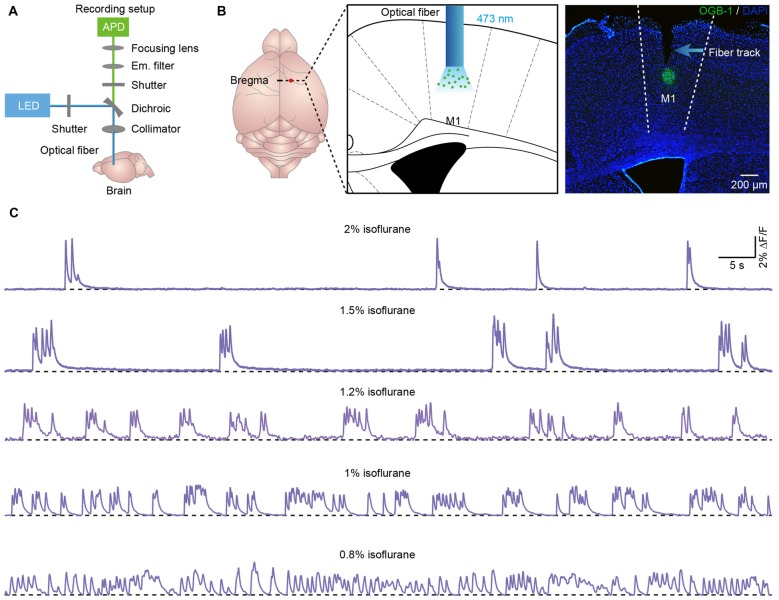
**Population cortical Ca^2+^ transients in anesthetized mice. (A)** Scheme of the optical fiber recording setup. **(B)** Left panel, schematic showing the tip of the optical fiber (diameter: 200 μm) implanted in layer 5 of the primary motor cortex (M1) stained with OGB-1AM. Right panel, *post hoc* fluorescence image of a coronal brain slice stained with OGB-1AM in layer 5 of the M1. **(C)** Examples showing the population Ca^2+^ transients in the M1 at different levels of anesthesia. The concentrations of isoflurane are indicated above each trace.

A custom-built fiber setup was used for the neuronal Ca^2+^ signal measurements (model “FiberOptoMeter v1.0,” Suzhou Institute of Biomedical Engineering and Technology; Figure 1A). The Ca^2+^ indicator Oregon green 488 BAPTA-1 (OGB-1) was excited at 488 nm by a solid-state laser. The light intensity was approximately 0.22 mW/mm^2^ at the tip of the fiber. The fluorescence emission was detected with an avalanche photodiode (Si APD, S2382, Hamamatsu Photonics K.K., Japan). The specialized optic setup was designed to accommodate the simultaneous recording of Ca^2+^ signals. The laser light on/off control and the data acquisition were managed using self-customized software on the LabVIEW platform (National Instruments, Austin, TX, USA).

### Fluorescent Ca^2+^ Indicator Staining

The animals were anesthetized with 1.5% isoflurane in pure O_2_ and then placed in a stereotactic head frame on a heating pad, where the animals were anesthetized with isoflurane until the surgery ended. The eyes were protected by ophthalmic ointment to prevent drying. A small craniotomy (0.5 × 0.5 mm) was made above the cortical area after removing the hair and skin. The coordinates of the craniotomy were as follows: for the M1 (from bregma): AP 0 mm, ML 1 mm (relative to midline); for the V1: AP 3 mm, ML 2.5 mm. A glass micropipette with a tip diameter of approximately 10 μm was placed directly above the skull and filled with OGB-1AM solution. Approximately 80–100 nl of solution was injected into the tissue at a depth of 600 μm (from the cortical surface). Following each injection, the pipette was kept in place for an additional 5 min before being slowly withdrawn.

### Optical Fiber Recordings in Anesthetized Mice

Approximately 30 min after dye application, a 200-μm-diameter optical fiber with a numerical aperture of 0.48 (Doric Lenses, Quebec City, QC, Canada) was inserted into the stained region with a micromanipulator. The fiber was glued into a short cannula (ID 0.51 mm, OD 0.82 mm) to maintain stability. To provide maximal fluorescence intensity, the fiber was advanced typically up to 550 μm below the cortical surface. At the beginning, the anesthesia level was adjusted to 2%. After the animals had adapted for 20 min and their respiration rates remained stable, the recording experiments began. After continuous recording for approximately 10 min, anesthesia was adjusted to a lower level. We then recorded again for another 10 min as described above. Recordings were obtained from each mouse under 2–3 anesthesia levels.

### Optical Fiber Recordings in Freely Moving Mice

After obtaining recordings from mice under anesthesia, the cannula and skull were fixed together using dental cement. After 20 min of solidification, the mice were moved back to their original house. Following recovering for approximately 2 h, the mice were placed into a white rectangular box (29 × 17 cm) in which they could move freely. A camera was placed just above the box and recorded the movements of the mice. Neuronal Ca^2+^ signals and locomotion were recorded simultaneously while the animals were freely moving. Every mouse was continuously recorded from approximately 40 min to 1 h. The Ca^2+^ transients were sampled at 2000 Hz with customized acquisition software based on the LabVIEW platform (National Instruments, Austin, TX, USA). The videos were recorded at 30 Hz at a spatial resolution of 1280 × 720 pixels (Aigo AHD-X9, China). This frame rate may lead to a maximal system error of 33 ms when we calculated the response latency. All the Ca^2+^ transients and behavior videos were synchronized offline using event marks.

### Histology and Fluorescence Imaging

To document the OGB-1 staining and confirm the relative position of the optical fiber, all recorded mice were perfused transcardially with 4% paraformaldehyde in phosphate-buffered saline (PBS) after the experiments. Brain samples were dehydrated with 15% sucrose in PBS for 24 h. Then, the brain samples were sectioned into 30-μm-thick slices, and 4′,6-diamidino-2-phenylindole was used to stain nuclei. Images were acquired using a fluorescence microscope and a 4× objective with a numerical aperture of 0.13.

### Data Analysis and Statistics

Ca^2+^ transients were acquired at a sampling rate of 2000 Hz after being converting into electrical signals through the Si APD. The data were low-pass filtered with a Savitzky-Golay finite-impulse response smoothing filter with 50 side points and a polynomial order of 3. Then, ΔF/F = (*f* − *f*_baseline_)/*f*_baseline_, relative fluorescence changes, were calculated as Ca^2+^ transients, where the *f*_baseline_ was the baseline level of fluorescence determined during the current recording period of the test. Ca^2+^ transients were automatically detected with a template-matching algorithm, taking into account the properties of rise and decay times of the Ca^2+^ signals. A Ca^2+^ transient was accepted as a signal when its amplitude was greater than three times the standard deviation of the noise band.

The mouse movement was calculated by the change in video clip image relative to its body size. Image frames from the video clip were converted into binary format to get the mouse shape according to its image intensities. A logical “OR” operation was performed between two consecutive frames to get the pixel size of absolute change of the mice. The ratio of such image change relative to the mouse body size, which was calculated beforehand, was defined as the mouse movement. This scoring procedure cannot differentiate the head movement from the general movement. Therefore, the head movements in the last figure were identified by eyes.

Statistical analysis was conducted in MATLAB (The MathWorks, Inc., Natick, MA, USA). For all statistical tests, significance was measured against an alpha of 0.05. The level of *p* < 0.05 was considered significant.

## Results

### Population Cortical Ca^2+^ Transients in Anesthetized Mice

We applied the optical fiber-based approach to record population Ca^2+^ activity in cortical neurons stained with the synthetic Ca^2+^ indicator OGB-1AM. We used a previously described fiber recording device (Adelsberger et al., 2005; Grienberger et al., 2012) that allows the excitation of OGB-1AM and the collection of emitted light (Figure 1A). We used the multicell bolus loading procedure to stain neurons located in layer 5 with OGB-1AM (Stosiek et al., 2003).

**Figure 2:**
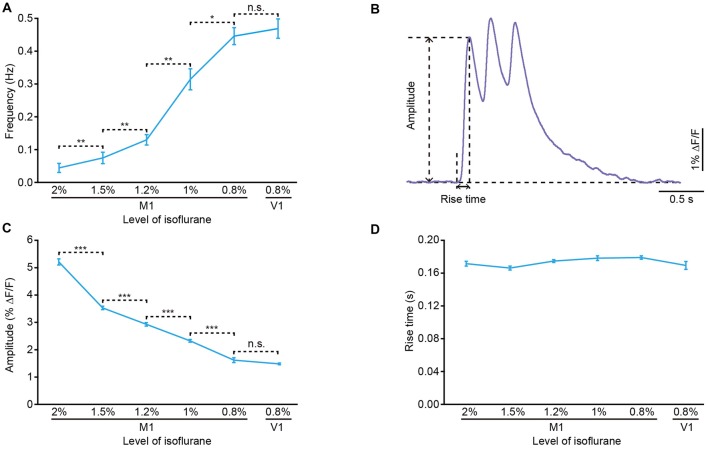
**Properties of the population cortical Ca^2+^ transients in anesthetized mice. (A)** Frequency of the population Ca^2+^ transients at different anesthesia levels in M1 and primary visual cortex (V1) (*n* = 7 and 7 mice, respectively; Wilcoxon rank-sum test, 2% vs. 1.5%, **p = 0.006; 1.5% vs. 1.2%, **p = 0.006; 1.2% vs. 1%, **p = 0.001; 1% vs. 0.8%, *p = 0.03; 0.8% (M1) vs. 0.8% (V1), *p* = 0.51). **(B)** Example of a Ca^2+^ transient for amplitude and rise time analysis. **(C)** Amplitude of the population Ca^2+^ transients at different anesthesia levels in M1 and V1 (*n* = 7 and 7 mice, respectively; Wilcoxon rank-sum test, 2% vs. 1.5%, ***p < 0.001; 1.5% vs. 1.2%, ***p < 0.001; 1.2% vs. 1%, ***p < 0.001; 1% vs. 0.8%, ***p < 0.001; 0.8% (M1) vs. 0.8% (V1), *p* = 0.88). **(D)** Rise time of the population Ca^2+^ transients at different anesthesia levels in M1 and V1 (*n* = 7 and 7 mice, respectively; Kruskal-Wallis test, *χ*^2^ = 5.86, *p* = 0.32). Values are the mean ± SEM.

Approximately 10 min after dye injection, we implanted an optical fiber with a diameter of 200 μm above the stained cortical area (Figure 1B). Similar to the previous work (Stroh et al., 2013), we detected slow oscillation-associated population Ca^2+^ transients under isoflurane anesthesia. Figure 1C shows examples of Ca^2+^ transients recorded in the M1 at different isoflurane levels (ranging from 0.8% to 2%). The quantitative analysis indicated that the population Ca^2+^ activity was strongly dependent on the level of anesthesia. In M1, the frequency increased from 0.04 ± 0.01 Hz to 0.45 ± 0.03 Hz with a decrease in the isoflurane concentration (Figure 2A). However, the amplitude (ΔF/F) was reduced from 5.21 ± 0.11 ΔF/F to 1.6 ± 0.08 ΔF/F when the isoflurane level was reduced from 2% to 0.8% (Figures 2B,C). In contrast, the rise time of the Ca^2+^ transients remained stable and was not affected by changes in the anesthesia level (Figure 2D; *p* = 0.32, *n* = 7 mice, Kruskal-Wallis test). In addition, the recordings of population activity in V1 revealed that no difference was found between M1 and V1 in frequency, amplitude and rise time of the Ca^2+^ transients at the same anesthesia level (Figures 2A,C,D; *p* > 0.05 for all the three parameters, *n* = 7 mice for V1).

### Population Ca^2+^ Transients in the M1 of Freely Moving Mice

**Figure 3:**
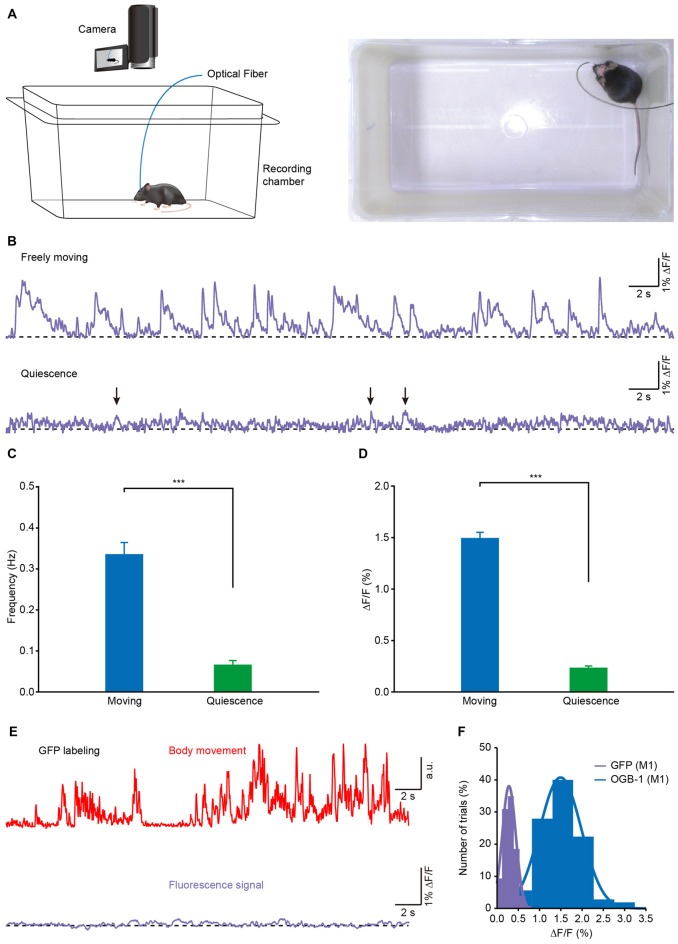
**Population Ca^2+^ transients of the M1 in freely moving and resting (quiescent but not sleeping) states. (A)** Left panel, scheme of the recording setup where Ca^2+^ transients and behavior were recorded simultaneously. Right panel, the actual recording condition. **(B)** Ca^2+^ transients of the M1 in freely moving and resting (but not sleeping) states. **(C)** Comparison of population Ca^2+^ transient frequencies in the M1 in freely moving and resting states in the first min after the mice were placed in the box (*n* = 8 mice; Wilcoxon signed-rank test, ***p < 0.001). **(D)** Comparison of population Ca^2+^ transient amplitudes in the M1 in freely moving and resting states (*n* = 8 mice; Wilcoxon signed-rank test, ***p < 0.001). **(E)** Example showing body movements (red) and simultaneously-recorded fluorescence (purple) from a green fluorescent protein (GFP) transgenic mouse during freely moving state. **(F)** Distribution of the amplitudes of OGB-1 and GFP fluorescence. Both fit Gaussian distributions and the mean values were 0.3% ΔF/F and 1.5% ΔF/F, respectively. Values are the mean ± SEM.

To investigate the correlation between the population Ca^2+^ transients and the body movements, we recorded Ca^2+^ activities in layer 5 neurons of the M1 in freely behaving mice in a white, opaque, rectangular chamber (Figure 3A). Mouse behavior was recorded with a camera that was placed above the recording chamber. The recordings were performed at least 2 h after anesthesia was ended. Figure 3B shows a representative recording of Ca^2+^ transients obtained from one mouse in both freely moving (upper) and resting (quiescent, but not sleeping; lower) states. In this example, when the mouse was moving freely, Ca^2+^ transients were observed with a high frequency, while almost no transients were observed in the resting states. On average, both the frequency (Figure 3C; moving: 0.32 ± 0.04 Hz vs. resting: 0.08 ± 0.01 Hz; *p* < 0.001, *n* = 8 mice, Wilcoxon signed-rank test) and amplitude (Figure 3D; moving: 1.51% ± 0.06% ΔF/F vs. resting: 0.24% ± 0.02% ΔF/F; *p* < 0.001, *n* = 8 mice, Wilcoxon signed-rank test) of the Ca^2+^ transients in the moving states were significantly higher than those in resting states. As a control, these signals were not seen in the mice whose layer 5 neurons expressed green fluorescent protein (GFP; Figures 3E,F).

**Figure 4:**
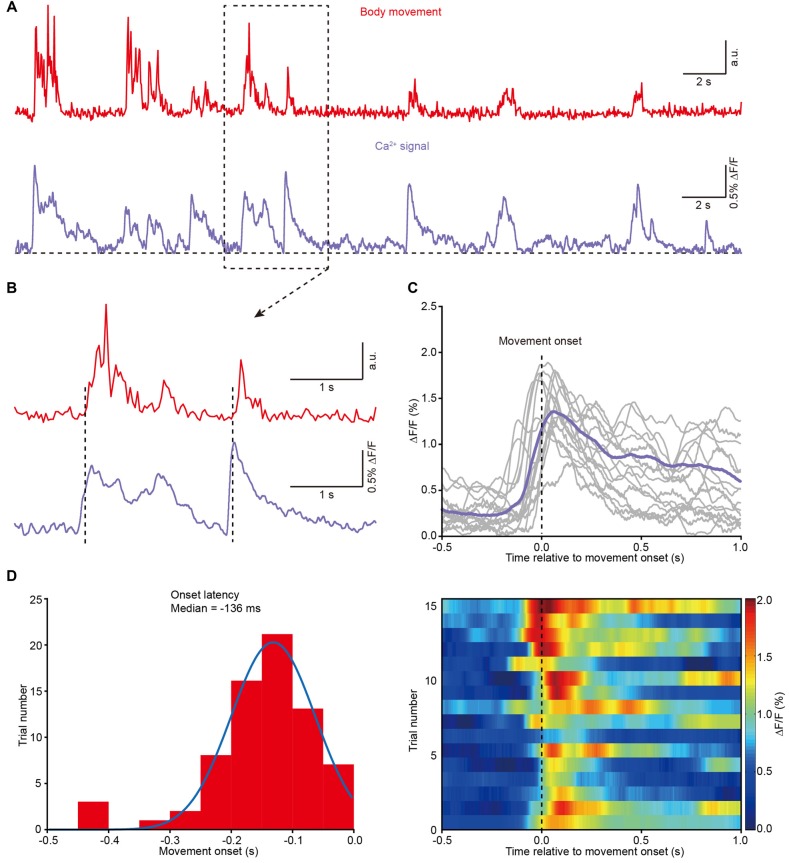
**Population Ca^2+^ transients in the M1 precede the onset of body movement. (A)** Example showing the body movements (red) and the related Ca^2+^ transients (purple) from a freely moving mouse. **(B)** Higher magnification of the dashed line box from panel **(A)**. **(C)** Top panel, 16 trials from eight different mice and their average (purple) showing the relationship between movement onset and Ca^2+^ transients. Bottom panel, color-coded intensity of Ca^2+^ transients from different trials. **(D)** Distribution of onset latencies between body movements and Ca^2+^ transients.

Next, we analyzed the correlation between body movement and the Ca^2+^ transients. We found that body movement (Figure 4A, upper) was always associated with Ca^2+^ transients in layer 5 neurons of the M1 (Figure 4A, lower). A closer analysis indicates that the Ca^2+^ transients preceded the onset of movement and were maintained throughout the entire process of each movement (see two examples in Figure 4B and the superimposition of 16 trials in Figure 4C). Across all the recordings (8 mice), the latency of the Ca^2+^ transients to the onset of movement fit a Gaussian distribution, and the median value was approximately −136 ms (Figure 4D).

### Population Ca^2+^ Transients in the V1 Precede the Onset of Body Movement

**Figure 5:**
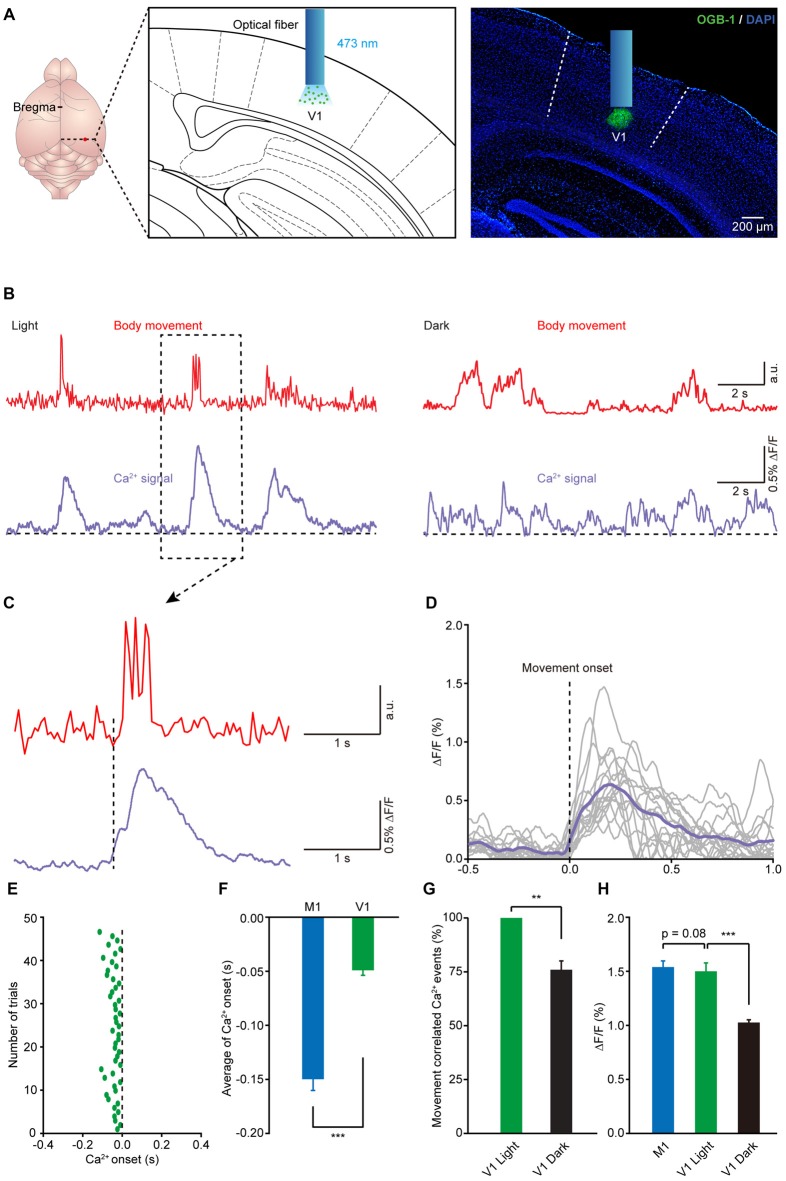
**Population Ca^2+^ transients in the V1 precede the onset of body movement. (A)** Left panel, schematic showing the optical fiber tip implanted above the layer 5 of the V1 stained with OGB-1. Right panel, *post hoc* fluorescence image of a coronal brain slice labeled with OGB-1 in layer 5 (green) of the V1. A blue bar indicates the position of an optical fiber. **(B)** Example showing the body movements (red) and the related Ca^2+^ transients (purple) in the V1 under both light (left) and dark (right) conditions. **(C)** Higher magnification of a dashed line box from **(B)**. **(D)** Sixteen single trials and their average (purple) showing the relationship between movement onset and Ca^2+^ transients in the V1. **(E)** Distribution of onset latency from 48 trials in eight mice. **(F)** Comparison of onset latencies of population Ca^2+^ transients in the M1 and V1 (*n* = 8 and 7 mice, respectively; Wilcoxon rank-sum test, ***p < 0.001). **(G)** Comparison of the movement-correlated Ca^2+^ events between light and dark conditions in V1 (*n* = 7 and 6 mice, respectively; Wilcoxon rank-sum test, **p < 0.01). **(H)** Comparison of population Ca^2+^ transient amplitude in M1, V1 (light) and V1 (dark) (*n* = 8 and 7 mice, respectively; Wilcoxon rank-sum test, M1 vs. V1 (light), *p* = 0.0854; *n* = 7 and 6 mice, respectively; Wilcoxon rank-sum test, V1 (light) vs. V1 (dark), ***p < 0.001). Values are the mean ± SEM.

It is well known that animals generally need to receive visual stimuli to guide and orient themselves during locomotion (Marigold, 2008). Recent studies have also reported that the activities of neurons in the visual cortex can be regulated by locomotion (Niell and Stryker, 2010; Keller et al., 2012; Ayaz et al., 2013). Thus, we wondered whether there were movement-related Ca^2+^ signals in the V1 of mouse during movement. Using the same approach, we recorded Ca^2+^ activities in layer 5 in the V1 in freely moving and resting states (Figure 5A). As expected, we found reliable movement-related population Ca^2+^ transients in layer 5 of the V1 (Figure 5B). Similar to the results observed in the M1, the onset of the Ca^2+^ transients slightly preceded the onset of impending movements and then persisted throughout the entire duration of the subsequent movement (see one example in Figure 5C and the superimposition of 14 trials in Figure 5D). Interestingly, the latency of the Ca^2+^ transients to the onset of movement was approximately −50 ms, which was later than the movement-related population Ca^2+^ transients in the M1 (Figures 5E,F; M1: 149.7 ± 10.3 ms vs. V1: 50.0 ± 4.7 ms, *n* = 8 and 7 mice, respectively; Wilcoxon rank-sum test). In addition, when we compared the amplitude of the population Ca^2+^ transients in the M1 and V1, there was no significant difference (Figure 5H; *p* = 0.085, *n* = 8 and 7 mice, respectively; Wilcoxon rank-sum test). Furthermore, we found that, compared to that under light conditions, in the dark both the amplitude and the correlation of movements and Ca^2+^ signals significantly decreased (Figures 5B,G,H).

### Population Ca^2+^ Transients of the M1 and V1 were Highly Correlated with Head Movement during Locomotion

**Figure 6:**
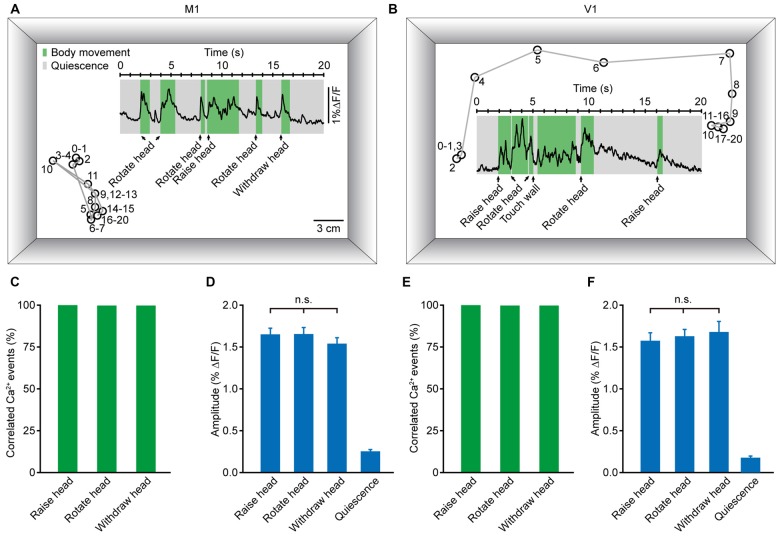
**Population Ca^2+^ transients in the M1 and V1 were highly correlated with head movements in freely moving mice. (A,B)** Examples showing the trial-by-trial correlation between head movements and population Ca^2+^ transients in the M1 **(A)** and V1 **(B)** in two different mice. Circles and gray lines indicate the position (head) and trajectory of the mouse, respectively; the large rectangular box indicates the recording chamber. **(C,E)** Ca^2+^ transients correlated with three different head movements: head raising, rotation and withdrawal in the M1 **(C)** and V1 **(E)**. **(D,F)** Comparison of population Ca^2+^ transient amplitudes of the three head movements in the M1 **(D)** and V1 **(F)** (M1: *n* = 8 mice, Kruskal-Wallis test, *χ*^2^ = 5.421, *p* = 0.066; V1: *n* = 7 mice, Kruskal-Wallis test, *χ*^2^ = 3.423, *p* = 0.181).

To accurately generate goal-directed movements, one must acquire essential information about position and orientation. Visual and non-visual sources of information together contribute crucially to orientation. During the process of generating body movements, head movement is a critical factor in the perception of orientation (Frissen et al., 2011; Yoder and Taube, 2014). As a self-motion cue, head movement can be used accurately to update the perceived orientation (Sun et al., 2004a,b; Siegle et al., 2009). Thus, we hypothesized that head movement may be closely associated with neuronal activity in both M1 and V1. Figures 6A,B display two examples of population Ca^2+^ transients related to head movements, which were obtained in layer 5 neurons of the M1 (Figure 6A) and V1 (Figure 6B), respectively. Here, we analyzed three kinds of head movements: raising, rotation and withdrawal. The correlated Ca^2+^ events were detected in a time window of 500 ms before the onset of movement. When the signal peak was higher above three times of standard deviation of the baseline, it was defined as a movement-correlated Ca^2+^event. We observed that the occurrence of each kind of head movement was always associated with population Ca^2+^ transients in both the M1 and V1. Across all recordings, the population Ca^2+^ transients were 100% correlated with head movements (Figures 6C,E). Next, we determined whether there were differences among the Ca^2+^ transient amplitudes of these three head movements. We found no significant differences in the M1 (Figure 6D; raising: 1.65 ± 0.07% ΔF/F, rotation: 1.65 ± 0.08% ΔF/F, withdrawal; 1.55 ± 0.07% ΔF/F; *p* = 0.066, *n* = 8 mice, Kruskal-Wallis test) or the V1 (Figure 6F; raising: 1.59 ± 0.08% ΔF/F, rotation: 1.63 ± 0.08% ΔF/F, withdrawal: 1.68 ± 0.13% ΔF/F; *p* = 0.181, *n* = 7 mice, Kruskal-Wallis test). In contrast, almost no Ca^2+^ transients were observed during resting (quiescent) states (Figures 6D,F).

## Discussion

In this study, we applied an optical fiber-based Ca^2+^ recording approach to monitor local population Ca^2+^ activity in layer 5 neurons in the M1 and V1 of freely moving mice. In layer 5 neurons of the M1, we found population Ca^2+^ transients frequently occurring in both anesthetized and freely moving states. However, we observed only a very small number of signals when mice were still (resting but not sleeping). During locomotion, we found that Ca^2+^ transients reliably occurred approximately 100 ms before the onset of movement and then persisted throughout the whole process of each movement. This activity was similar to a previously defined activity in the monkey motor cortex, referred to as perimovement activity (Churchland et al., 2006, 2010; Kemere et al., 2008; Zimnik et al., 2015). Perimovement activity is defined as neuronal activity occurring approximately 100 ms before, during and just after the movement and was initially found using electrophysiological recordings. This neuronal activity was highly correlated with body movement in our recordings (Figure 6D). Moreover, we did not observe “preparatory activity,” which occurs much earlier than perimovement activity, approximately 900 ms before the onset of movement. Preparatory activity was thought to be related to motor planning (Churchland et al., 2010; Erisken et al., 2014; Flash and Bizzi, 2016) and was recently described in the mouse premotor cortex (anterior lateral motor cortex) but not in the M1.

Similar to the results in the M1, we found that population Ca^2+^ transients preceding impending movement also occurred in the layer 5 neurons of the V1. Interestingly, such activity in the V1 started ~100 ms later than that in M1. Previous studies have reported this preceding activity in upper layers as well as in deep layers using electrophysiological recordings in the V1 (Ayaz et al., 2013; Erisken et al., 2014; Vinck et al., 2015). Recent work has suggested that similar activity can originate from inhibitory interneurons (Polack et al., 2013; Reimer et al., 2014; Pakan et al., 2016), especially parvalbumin-positive interneurons (Polack et al., 2013; Pakan et al., 2016). In addition, this activity is probably the result of a combination of visual- and motor-related inputs (Ayaz et al., 2013; Erisken et al., 2014), which may reflect the interaction between animal locomotion and its environment. Therefore, this signal processing in the visual cortex may be essential for estimating and correcting information relevant for goal-directed navigation (Keller et al., 2012; Ayaz et al., 2013). Future work is needed to investigate the specific role of V1 signals in the generation of locomotion.

Population activity plays an important role in many brain functions, such as information processing, learning, memory and behavior. For example, extensive studies have focused on slow oscillations, i.e., slow population activity at a frequency less than 1 Hz mainly occurring during sleep and anesthesia (Steriade et al., 1993a,b,c; Brustein et al., 2003). Other types of population activity, such as theta and gamma waves, have also been described in different brain regions and thought to be relevant for brain functions (Seager et al., 2002; Nokia et al., 2008; Carr et al., 2012), such as attention (Ayaz et al., 2013; Başar et al., 2013; Clayton et al., 2015). The methods used to study this population activity were mainly dependent on electrophysiological recordings, such as local field potential recordings with a high temporal resolution. Here, we recorded the population activity from a spatially clustered group of neurons using an optical fiber-based approach. By locally injecting a small amount of Ca^2+^ dyes, we could achieve a recording of activity in a highly restricted area approximately 300–500 μm in diameter. In many other studies (Lütcke et al., 2010; Marshall et al., 2016), the use of genetically encoded Ca^2+^ or voltage sensors allowed the activity of cell type-specific neural populations to be recorded during free behavior. Using this simple but efficient approach, we studied locomotion-related population signals of neurons in deep layers of both the M1 and V1. In the near future, the application of endoscope-based imaging techniques with cellular resolution (Jung et al., 2004; Flusberg et al., 2005; Ziv et al., 2013) will provide more detailed information about the properties and sources of these locomotion-related signals.

In summary, our results illustrate that the optical fiber-based approach is an efficient method for monitoring cortical Ca^2+^ activity in freely behaving animals. Using this method, we provide insights into body movement-related population neuronal activity in both the M1 and V1, which could be the first step toward understanding cortical information processing during locomotion.

## Author Contributions

QZ, JYao, YG, HJ, JYan, ZF, WL and XC contributed to the design of the study and interpretation of the data. QZ, JYao, YG, HQ, JZ, JP and HJ performed the experiments and acquired the data. QZ, SL, JG, XL, WJ and HQ processed and analyzed the data. QZ, ZF, WL and XC wrote the manuscript with help from all the other authors.

## Conflict of Interest Statement

The authors declare that the research was conducted in the absence of any commercial or financial relationships that could be construed as a potential conflict of interest.
